# Melanotic neuroectodermal tumor of infancy in the mandible: A case report

**DOI:** 10.1097/MD.0000000000028001

**Published:** 2021-12-17

**Authors:** Ryoko Takeuchi, Akinori Funayama, Yohei Oda, Tatsuya Abé, Manabu Yamazaki, Satoshi Maruyama, Takafumi Hayashi, Jun-ichi Tanuma, Tadaharu Kobayashi

**Affiliations:** a Division of Reconstructive Surgery for Oral and Maxillofacial Region, Faculty of Dentistry & Graduate School of Medical and Dental Science, Niigata University, Niigata, Japan; b Division of Oral Pathology, Niigata University, Faculty of Dentistry & Graduate School of Medical and Dental Science, Niigata University, Niigata, Japan; c Oral Pathology Section, Department of Surgical Pathology, Niigata University Hospital, Niigata, Japan; d Division of Oral and Maxillofacial Radiology, Faculty of Dentistry & Graduate School of Medical and Dental Science, Niigata University, Niigata, Japan.

**Keywords:** case report, fontana-masson, mandible tumor, marginal mandibulectomy, melanotic neuroectodermal tumor of infancy

## Abstract

**Rationale:**

Melanocytic neuroectodermal tumor of infancy (MNTI) is a rare benign pigmented neoplasm that arises from the neural crest and has an aggressive growth pattern. It is predominantly seen in infants under 1 year of age, and the most common site of involvement is the maxilla. The currently accepted treatment is removal by surgical resection. Herein, we report a case of MNTI that involved the anterior alveolar ridge of the mandible in a 6-month-old infant.

**Patient concerns:**

A case of a 6-month-old male child with a huge mass in the anterior alveolar ridge of the mandible.

**Diagnosis:**

The tumor was diagnosed using histopathological and immunohistochemical techniques on the biopsy specimen obtained following incisional biopsy. Based on the findings, a final diagnosis of MNTI was established.

**Interventions:**

Radical resection of the tumor was performed, after determining the extent of resection by referring to the mandibular 3D model created using the pre-operative CT data.

**Outcomes:**

The postoperative course was uneventful, and no recurrence has been observed to date for more than 4 years after surgery.

**Lessons:**

This case emphasizes that early diagnosis and radical surgery are critical to the effective treatment, as MNTI exhibits rapid and destructive growth. It also requires careful and close follow-up because of high recurrence rates.

## 1 Introduction

Melanocytic neuroectodermal tumor of infancy (MNTI) is a rare benign pigmented neoplasm of neural crest origin, which was first described by Krombecher as congenital melanocarcinoma in 1918.^[1]^ Although MNTI is a benign neoplasm, it is considered to be a locally aggressive tumor because of its high capacity to proliferate and invade surrounding tissues with little distant metastasis.^[2–4]^ MNTI is commonly seen in infants under 1 year of age and is predominant in males. The most common site of occurrence is the maxilla, with approximately two-thirds of lesions occurring there. However, there are reports that MNTI can also involve the mandible, skull, brain, epididymis, and mediastinum.^[5]^ Surgical resection is the conventional definitive treatment for this tumor. Due to the relatively high recurrence rate, MNTI should be followed carefully after surgery. In this paper, we describe a case of MNTI involving the mandibular anterior alveolar ridge of a 6-month-old infant.

This study was approved by ethics committee review board of Niigata University (Approval number: 2021–0169, Applicant: Akinori Funayama). Written informed consent was obtained from the patient's family for publication of the case details and accompanying images.

## 2 Case report

A 6-month-old male child was referred to our hospital with a mass on the anterior alveolar ridge of the mandible. The patient's parents noticed this mass at the age of 4 months, which rapidly increased in size thereafter.

**Figure 1:**
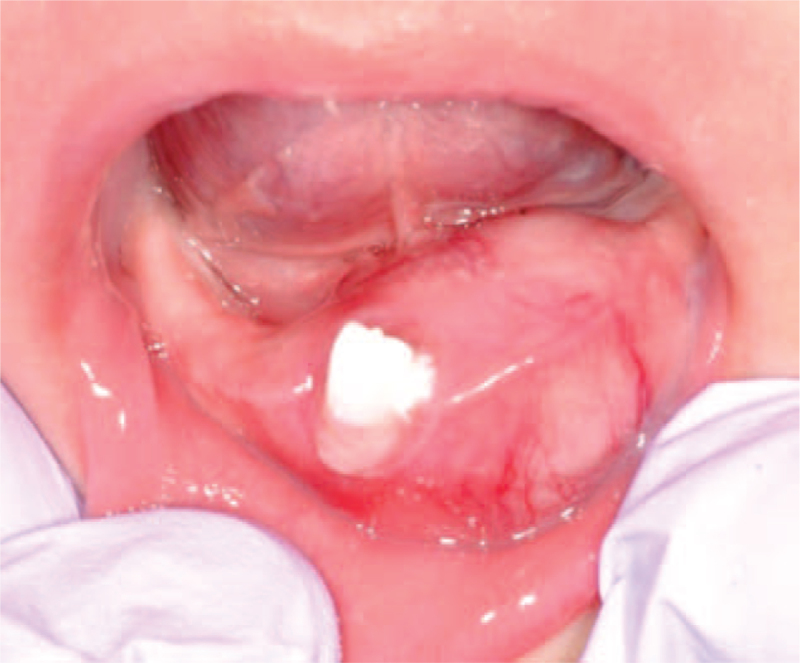
Intraoral photography at initial examination. A well-defined spherical mass (30 × 20 × 20 mm) of normal mucosal color seen on the mandibular alveolar ridge extending from the right mandibular deciduous canine to the left mandibular first deciduous molar. The left mandibular deciduous central incisor can be seen attached to the surface of the tumor.

At his first visit to our hospital, his face was symmetrical, but the lower lip was protruded. A relatively well-defined spherical mass (30 × 20 × 20 mm) was located on the mandibular alveolar ridge extending from the right mandibular deciduous canine to the left mandibular first deciduous molar (Fig. 1). The mass had normal mucosal color, was elastic hard and non-fluctuant with the left mandibular deciduous central incisor attached to the surface of the tumor.

**Figure 2:**
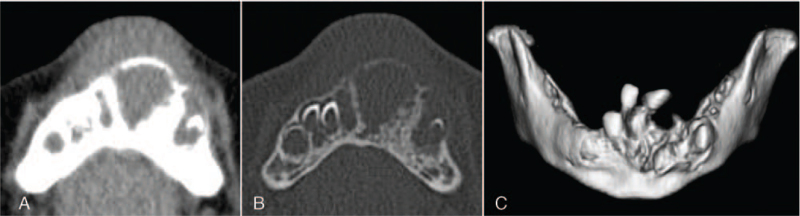
Axial CT image showing a large mass in the mandibular alveolus. Soft tissue window. Bone window. 3D reconstruction of CT image showing numerous tooth germ displacements.

**Figure 3:**
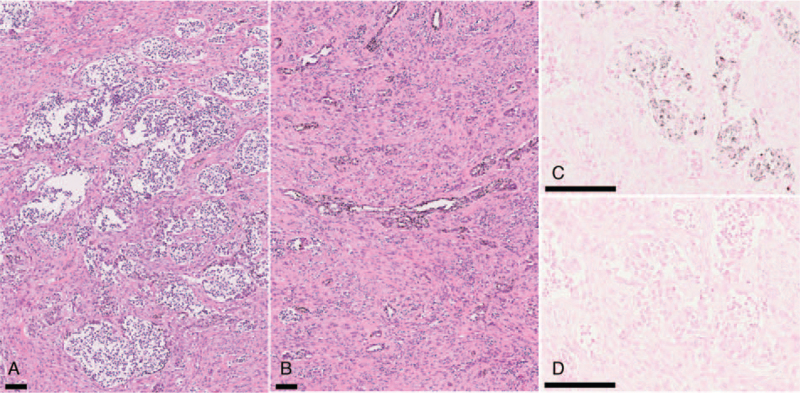
Routine staining of biopsy specimens. (A) Hematoxylin-eosin (HE) staining shows the large and small round cells proliferating in the relatively dense fibrous connective tissue, forming alveolar or cord-like structures. (B) HE staining showing a tubuloglandular-like structure consisted of the epithelioid tumor cells with melanin pigmentation. (C) Fontana-Masson staining for detection of melanin pigment. (D) The bleaching method for melanin with potassium permanganate–oxalic acid. Scale-bar 100 μm.

**Figure 4:**
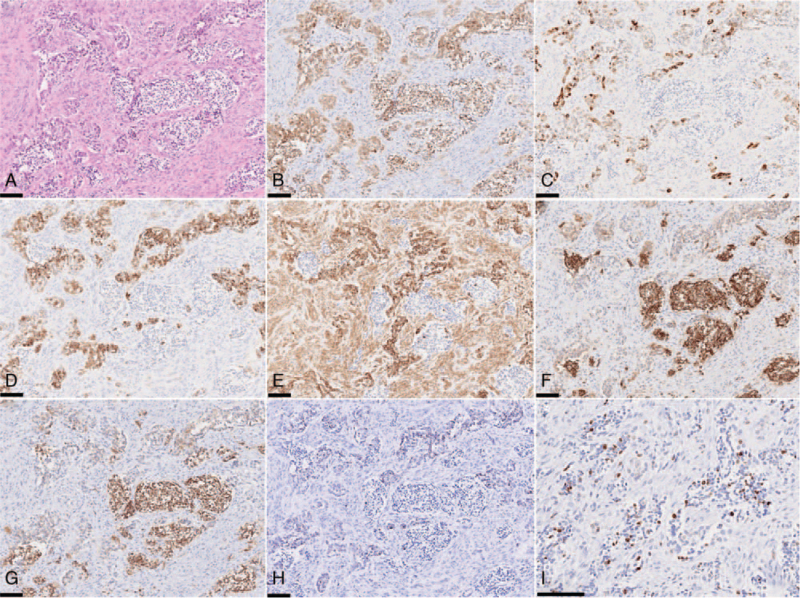
Immunohistochemistry findings. (A) HE. (B) NSE. (C) AE1/AE3. (D) HMB-45. (E) Vimentin. (F) CD56. (G) Synaptophysin. (H) S-100. (I) Ki-67. Scale-bar 100 μm.

Computed tomography (CT) showed a relatively uniform multicentric osteolytic lesion extending from the right mandibular deciduous central incisor to the left mandibular deciduous canine with expansion of the surrounding bone and numerous displaced tooth germs. The density of the lesion mass was lower than that of the muscle, and the boundary with cancellous bone was irregular (Fig. 2). Histopathological findings of the biopsy specimen showed that the biphasic tumor cells consisted of large melanin-containing epithelioid cells and small neuroblast-like cells forming a solid alveolar pattern (Fig. 3A) and a gland-like pattern (Fig. 3B). The large tumor cells had a slightly eosinophilic cytoplasm and an oval bright nucleus with small but distinct nucleoli. These were occasionally positive for melanin pigment, which was visualized in the form of black granules on Fontana-Masson staining (Fig. 3C) and were bleached with potassium permanganate–oxalic acid (Fig. 3D). The small tumor cells had a scanty cytoplasm and a slightly deeply stained round nucleus, often showing detached clusters. A small number of mitotic figures, and a low degree of cellular polymorphism were observed. Immunohistochemistry revealed that the large and small tumor cells were positive for neuron-specific enolase (Fig. 4A & B). The large tumor cells stained positive for cytokeratin (AE1/AE3), human melanin black-45, and vimentin (Fig. 4C–E). The small tumor cells stained positive for CD56 and synaptophysin (Fig. 4F & G). The tumor cells were negative for S-100 (Fig. 4H) and chromogranin A. The MIB-1 proliferation index was >50% (Fig. 4I). Based on these findings, the tumor was finally diagnosed as MNTI.

**Figure 5:**
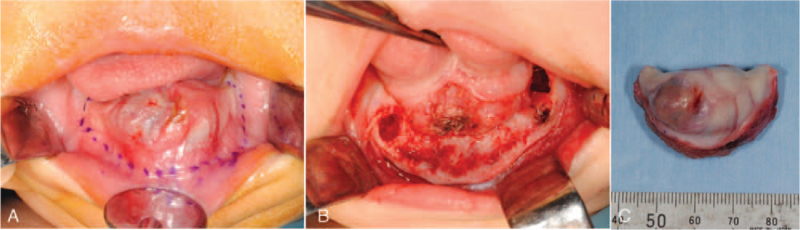
Marginal mandibulectomy. (A) Assumed excision line before the surgery. (B) Marginal mandibulectomy was performed. (C) Gross specimen with heterogeneity on complete resection.

The tumor continued to grow rapidly, and marginal mandibulectomy was performed under general anesthesia 28 days after the first visit to our hospital. The extent of resection was determined by referring to the mandibular 3D model which was created based on the CT data obtained before the surgery. Marginal mandibulectomy, approximately 5 mm lateral to the tumor, was successful in preserving the inferior margin of the mandible (Fig. 5). The mental foramen on the left side was included in the resection, and the foramen on the right side was preserved. All deciduous tooth germs were included in the excision, except for the right deciduous second molar. After rounding the sharp edges of the bone with a file, the raw surface of the mandible was completely closed by the surrounding soft tissue with help of polydioxanone monofilament synthetic absorbable suture. The postoperative course was uneventful, and oral intake of milk was started 3 days after the operation. On the 7th day after the surgery, the patient was discharged from the hospital with good general condition. At the time of discharge, he had no abnormal facial swelling or infection in the surgical wound.

Similar to biopsy specimens, histopathological findings of the excised mass showed that the tumor consisted of 2 types of cells: large polyhedral cells sometimes containing melanin granules and smaller round cells with hyperchromatic nuclei and scanty cytoplasm resembling lymphocytes. No nuclear atypia or mitosis was observed. Tumor cells were seen invading the surrounding tooth germs and bone.

**Figure 6:**
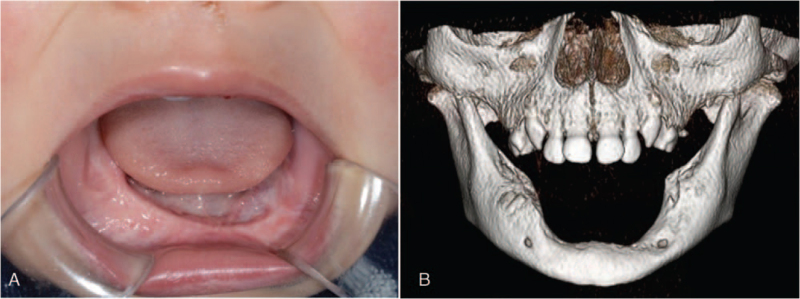
Postoperative state. (A) Intraoral photography. (B) 3D reconstruction of CT images 1 year after surgery revealed no evidence of recurrent tumor.

The patient is being closely followed up, and no evidence of recurrence or metastasis was observed on CT images taken 1 year after surgery (Fig. 6). In this case, stomatognathic functions such as eating, swallowing, and pronunciation were normal post-surgery, but occlusal reconstruction and recovery of masticatory function following removal of the mandibular dentition will be important therapeutic goals in the future.

## 3 Discussion

MNTI is a rare infantile neoplasm that has previously been described by various names such as melanotic progonoma, melanotic adamantinoma, melanotic hamartoma, melanoameloblastoma, pigmented epulis, pigmented adamantinoma, congenital melanocarcinoma, and retinal anlage tumor, because of its uncertain origin. However, Borello and Gorlin revealed that this tumor produces urinary vanillylmandelic acid (VMA), which identified the neural crest origin of the disease. They also reported that the tumor is biphasic, consisting of epithelioid melanogenic cells and neuroblast-like cells.^[6]^ Although VMA is useful for diagnosing tumors of neural crest origin, most patients with MNTI, including our previous patient, showed normal levels of VMA.^[7]^ Therefore, VMA seems to have no relationship with the biological behavior of MNTI.

The histological diagnosis of MNTI is relatively simple and rarely poses a diagnostic challenge if an appropriate tissue specimen is available for histopathological examination. The differential diagnosis of MNTI includes other neoplasms of infancy, especially neuroblastoma, Ewing sarcoma, and alveolar rhabdomyosarcoma.^[8]^ However, this case did not match any of the histopathological findings. Histopathologically, all MNTIs have biphasic morphology characterized by small primordial neuroblast-like cells and larger peripheral epithelioid cells containing melanin granules. Immunohistochemistry can be used as a diagnostic aid, where biopsy specimens reveal a neuroblast-like morphology and expression of neuronal markers such as neuron-specific enolase, synaptophysin, and chromogranin A can confirm the neuroblastic origin of the small cells.^[9]^ The large cells have a lightly eosinophilic or vacuolated cytoplasm, an oval bright nucleus with small nucleoli, and are of ectodermal origin, which can be confirmed by epithelial membrane antigen, cytokeratin, and HMB-45 positivity. This heterogeneity of cellular phenotypes is probably explained by the morphological features of the mesoderm and ectoderm displayed by neural crest cells at different stages of their ontogeny.^[5]^

More than 500 cases of MNTI have been reported worldwide by Rachidi et al. In addition to the 472 cases reported between 1918 and 2013 as a part of the largest retrospective study,^[5]^ we found 49 cases reported between January 2014 and June 2021 by searching PubMed.^[3,4,9–32,36–38]^ In a systematic review of 472 MNTI cases,^[5]^ most cases developed within 1 year of age, with more than 90% of cases occurring in the craniofacial region, such as the maxilla (60.3%), skull (18.1%), and mandible (10.3%). On the other hand, to summarize the reported cases since 2014, 27 (87.1%) cases of MNTI developed within 1 year of age, and 32 (65.3%) cases occurred in the maxilla, 4 (8.2%) in the mandible, and 1 (2%) in the hard palate, while 12 (24.5%) cases occurred outside the oral cavity, such as the cranial bone, middle ear, upper arm, fibula, and ovary.

MNTI is typically a benign lesion and presents as an enlarging, painless, and firm mass, often underlying an intact epithelial surface; however, it has a relatively high risk of recurrence, and peripheral lymph nodes may be involved. Metastasis to distant organs occurs in approximately 6.5% of patients.^[37,38]^ The definitive treatment for MNTI is usually surgical resection.^[33]^ Complete resection of the lesion is a particularly important factor for determining disease-free survival time. Chemotherapy may be effective in cases where complete resection of the lesion is difficult or where the tumor remains or metastasizes.^[3,29,38]^ To reduce the possibility of recurrence, it is necessary to include the adjacent teeth, bones, and soft tissues within the resection margin according to the extent of the tumor. Some authors argue that the surgically resected area should include a margin of 5 mm in healthy tissue.^[33–35]^ There is no obvious treatment strategy for metastatic and/or recurrent MNTI that is unresectable. In these cases, chemotherapy may be effective for treating large tumors and residual tumors.^[36,38]^ In a previous study, only 3 out of 249 cases of MNTI were treated with chemotherapy without surgery.^[5]^ However, the combination of surgery, chemotherapy, and radiotherapy can cause serious acute and late radiation injury in infant patients and requires careful consideration and restricted use. Recurrence rates for MNTI have been reported to be 15% to 27% for multiple reasons, including incomplete resection and the occurrence of multicentric tumors.^[2,5,8]^ In addition, cases of MNTI diagnosed before 2 months of age have the highest risk of recurrence and all cases of relapse occur within 6 months of surgery.^[5]^ We have also encountered a previous case in which a segmental mandibulectomy had to be performed because of recurrence, 1 month after the first surgery.^[7]^

Although definitive MNTI treatment guidelines have not been established, radical surgery with safety margins is the accepted current gold standard because of the rapid and destructive growth of the tumor into the surrounding tissues and the high propensity for recurrence. In addition, early diagnosis and regular follow-up are important. On the other hand, in the case of a large MNTI and extensive tissue loss, reconstruction of the maxillofacial region is essential, which can be very challenging in infants.^[37]^ In the present patient, marginal mandibulectomy based on appropriate early diagnosis was performed, including almost all deciduous and permanent tooth germs ranging from the mandibular anterior teeth to premolars, and no recurrence has been observed to date more than 4 years after surgery. In the future, occlusal reconstruction and recovery of masticatory function following surgery will be the crucial therapeutic goals.

## Author contributions

**Conceptualization:** Ryoko Takeuchi, Akinori Funayama, Takafumi Hayashi, Jun-Ichi Tanuma, Tadaharu Kobayashi.

**Data curation:** Ryoko Takeuchi.

**Formal analysis:** Ryoko Takeuchi, Akinori Funayama, Tatsuya Abé, Takafumi Hayashi, Tadaharu Kobayashi.

**Investigation:** Ryoko Takeuchi, Akinori Funayama, Yohei Oda, Tatsuya Abé, Manabu Yamazaki, Satoshi Maruyama, Takafumi Hayashi, Tadaharu Kobayashi.

**Methodology:** Ryoko Takeuchi, Akinori Funayama, Yohei Oda, Tatsuya Abé, Manabu Yamazaki, Satoshi Maruyama, Takafumi Hayashi, Jun-ichi Tanuma, Tadaharu Kobayashi.

**Project administration:** Tadaharu Kobayashi.

**Supervision:** Akinori Funayama, Jun-ichi Tanuma, Tadaharu Kobayashi.

**Validation:** Ryoko Takeuchi, Akinori Funayama, Yohei Oda, Tatsuya Abé, Manabu Yamazaki, Satoshi Maruyama, Takafumi Hayashi, Jun-ichi Tanuma, Tadaharu Kobayashi.

**Visualization:** Ryoko Takeuchi, Tatsuya Abé, Jun-ichi Tanuma, Tadaharu Kobayashi.

**Writing – original draft:** Ryoko Takeuchi.

**Writing – review & editing:** Ryoko Takeuchi, Akinori Funayama, Tadaharu Kobayashi.
